# Abnormal regional spontaneous neural activity and functional connectivity in thyroid-associated ophthalmopathy patients with different activity: a resting-state fMRI study

**DOI:** 10.3389/fneur.2023.1199251

**Published:** 2023-07-05

**Authors:** Mengda Jiang, Haiyang Zhang, Yuting Liu, Shuo Wu, Jialu Qu, Yan Tang, Yang Song, Yinwei Li, Jing Sun, Ling Zhu, Huifang Zhou, Xiaofeng Tao

**Affiliations:** ^1^Department of Radiology, Shanghai Ninth People’s Hospital, Shanghai Jiao Tong University School of Medicine, Shanghai, China; ^2^Department of Ophthalmology, Shanghai Ninth People’s Hospital, Shanghai Jiao Tong University School of Medicine, Shanghai, China; ^3^Shanghai Key Laboratory of Orbital Diseases and Ocular Oncology, Shanghai, China; ^4^Shanghai Jiao Tong University School of Medicine, Shanghai, China; ^5^Department of MR Scientific Marketing, Siemens Healthcare, Shanghai, China

**Keywords:** thyroid-associated ophthalmopathy, resting-state functional magnetic resonance, amplitude of low-frequency fluctuation, fractional amplitude of low-frequency fluctuation, functional connectivity

## Abstract

**Purpose:**

We aimed to evaluate the spontaneous neuronal activity and functional connectivity pattern variations using resting-state functional magnetic resonance imaging (rs-fMRI) measures, such as amplitude of low-frequency fluctuation (ALFF), fractional amplitude of low-frequency fluctuation (fALFF), and functional connectivity (FC), in patients with thyroid-associated ophthalmopathy (TAO).

**Method:**

A total of 24 active TAO patients, 26 inactive TAO patients, and 27 matched healthy controls (HCs) were included. First, ALFF and fALFF were used to detect local neural activity changes, the MRI data were analyzed, and regions with group differences were taken as seeds. Second, FC analysis was performed to explore the altered connection between seeds and other brain regions. A correlation analysis was performed to assess the relationship between functional brain activity and clinical indices and neuropsychiatric behaviors.

**Results:**

Compared to HCs, both active and inactive TAO patients exhibited significantly lower ALFF values in the right calcarine (Calcarine_R) and left postcentral gyrus (Postcentral_L). Active TAO patients also showed significantly higher ALFF values in the left caudate nucleus (Caudate_L) and increased fALFF values in the superior lobe of the right cerebellum (Cerebelum_Crus1_R). Moreover, both active and inactive TAO patients demonstrated decreased FC within the left postcentral gyrus (Postcentral_L) compared to HCs. Additionally, active TAO patients exhibited lower FC compared to inactive TAO patients. The ALFF values in the Calcarine_R of active TAO patients positively correlated with disease duration (r = 0.5892, *p* = 0.0049) and the Hamilton Anxiety Rating Scale (HARS; r = 0.5377, *p* = 0.0119). Furthermore, the ALFF value in the Calcarine_R of inactive TAO patients negatively correlated with visual functioning (r = −0.5449, *p* = 0.0072), while the ALFF values in the Caudate_L of active TAO patients positively correlated with visual functioning (r = 0.6496, *p* = 0.0014).

**Conclusion:**

We found that the Caudate_L and Cerebelum_Crus1_R related to motor control and coordination in active TAO patients exhibit significant compensatory mechanisms; whereas, the Calcarine_R and Postcentral_L related to visual and somatosensory cortices show varying degrees of impairment. Our findings complement the functional neural mechanism of TAO.

## 1. Introduction

Thyroid-associated ophthalmopathy (TAO), which is also known as Graves ophthalmopathy or thyroid eye disease, is a progressive autoimmune, inflammatory disease that mainly affects the orbit. TAO is the most prevalent autoimmune orbital disease in adults and it typically affects both orbits causing an asymmetrical appearance (1, 2). The most common ophthalmic symptoms in TAO patients include swelling around the eyes, highly raised eyelids, diplopia, exophthalmos, difficulty moving the eyes, and impaired vision (3, 4). TAO has a biphasic process—first, it has an active phase, which is characterized by orbital inflammation; next, it has an inactive phase, which is characterized by fibrosis (5). TAO patients in the active phase have inflammatory orbital tissue. Thus, they show a good response to anti-inflammatory treatment; however, rehabilitative surgery is suggested for patients in the inactive phase because of steroid resistance to interstitial fibrosis (6).

TAO can lead to long-lasting vision impairment and substantial facial deformities, which can significantly impact the patient’s overall well-being by limiting their daily activities, impairing their social interactions, and reducing their self-esteem (7, 8). A deep understanding of the neuropathological mechanisms involved in TAO and early identification to prevent associated neuropsychic dysfunctions is crucial.

Recently, advances in neuroimaging technology have allowed for better investigation of the underlying mechanisms of neuropsychiatric disorders. Particularly, resting-state functional magnetic resonance imaging (rs-fMRI) has emerged as a valuable tool for assessing the relationship between brain changes and various ophthalmological conditions (9–12). rs-fMRI can detect differences in the magnetic properties of oxyhemoglobin and deoxyhemoglobin, and thus, it generates blood-oxygen-level-dependent (BOLD) signals from different brain regions, even when the patient resting with closed eyes (13).

Zang et al. introduced a novel technique called analysis of amplitude of low frequency fluctuation (ALFF) for investigating brain function (14) in patients with attention deficit hyperactivity disorder. This technique measures the energy of low-frequency oscillations and detects spontaneous brain activity changes. Recently, Chen et al. (7) utilized ALFF to analyze brain activity in a small sample study on patients with TAO. They identified alterations in ALFF within the occipital lobe (left middle occipital gyrus, superior occipital gyrus, and cuneus), a brain region associated with vision. These findings suggest a possible disruption in visual function among individuals with TAO. Another study used ALFF to investigate TAO and found that TAO patients had significantly lower ALFF values in the right superior occipital gyrus and bilateral precuneus and higher ALFF values in the left cerebellum and left insula than healthy controls (HCs) had. These findings suggest that TAO leads to changes in neuronal activity in various brain regions, especially those involved in visual performance, emotional processing, and cognitive function (15).

Based on ALFF, Zou et al. (16) proposed fraction ALFF (fALFF), which can remove physiological noise effectively and has a higher sensitivity and specificity compared to ALFF. Thus far, few studies have utilized fALFF to investigate TAO. Zhu et al. (17) conducted a study on active TAO patients using fALFF and noticed increased fALFF values in the right temporal lobe and left cingulate gyrus and decreased fALFF values in the right calcarine, indicating impaired visual information processing and emotional and cognitive function in active TAO patients. However, previous studies that have used ALFF and fALFF to study TAO have had relatively small sample sizes and non-uniform voxel p-thresholds. Moreover, functional connectivity (FC) analysis assessed the spatial and temporal correlations and synchrony of the BOLD signals between anatomically distinct brain regions (18).

Herein, to explore the neural mechanism of TAO more comprehensively, we combined ALFF/fALFF and FC to quantify and compare local and global spontaneous neural activity in active and inactive TAO patients and HCs.

## 2. Materials and methods

### 2.1. Participants

Twenty-four active and 26 inactive TAO patients were included in this study. Twenty-seven HCs were matched for age, sex, and years of education with the TAO patients. All patients were recruited from the Department of Ophthalmology, Shanghai Ninth People’s Hospital, Shanghai Jiao Tong University School of Medicine, Shanghai, China, between October 2021 and January 2023. Bartley’s diagnostic criteria were applied for diagnosing TAO (19). The TAO activity was measured by clinical activity score (CAS) (20), and a CAS of ≥3/7 represented active TAO. The patients were divided into two groups–the active TAO group (CAS ≥ 3) and the inactive TAO group (CAS < 3). Participants were excluded if they had: (1) ocular symptoms or a history of diseases; (2) eye surgery; (3) psychiatric or neurological illness; (4) brain structural abnormalities; (5) ineligibility for MRI scanning; (6) low-quality images that would affect the accuracy of fMRI analysis; and (7) signs of dysthyroid optic neuropathy.

This study was approved by the Ethics Committee of Shanghai Ninth People’s Hospital, Shanghai Jiao Tong University School of Medicine (approval number: SH9H-2022-T229-2).

### 2.2. Questionnaire assessments

Before the MRI scan, we performed neuropsychological evaluations and quality of life (QoL) assessments using the English version of the Graves orbitopathy-specific questionnaire obtained from the European Group on Graves orbitopathy (EUGOGO) website and translated it for the TAO patients as done in the study by Lin et al. (21). The questionnaire comprises two subscales for life quality—visual function and appearance. Moreover, the 17-item Hamilton depression rating scale (HDRS) and the 14-item Hamilton anxiety rating scale (HARS) were used to evaluate depression and anxiety for all patients.

### 2.3. MRI acquisition

A 3.0 Tesla scanner (Magnetom Vida, Siemens, Erlangen, Germany) equipped with a 64-channel phase array head coil was used for the MRI scans. Head motion and scanning noise were reduced by using foam padding and earplugs. All patients were asked to close their eyes without falling asleep when undergoing the MRI scanning. The high-resolution sagittal structural T1-weighted images of the whole brain with the following parameters were obtained—repetition time = 2,400 ms, echo time = 2.4 ms, thickness = 0.8 mm, gap = 0 mm, acquisition matrix = 320 × 320, field of view = 256 × 256 mm^2^, flip angle = 8°, number of slices = 208, and voxel size = 1 × 1 × 1 mm^3^ for a total of 6 min and 52 s. Additionally, functional images of the whole brain with the following parameters were obtained—repetition time = 2,000 ms, echo time = 30 ms, thickness = 2.0 mm, gap = 0 mm, acquisition matrix = 78 × 78, field of view = 208 × 208 mm^2^, flip angle = 90°, number of slices = 72, in-plane resolution = 2 × 2 mm^2^, and 240 volumes with a total of 8 min 13 s.

### 2.4. Data processing

#### 2.4.1. Data preprocessing

All rs-fMRI data were preprocessed using DPASFA version 5.2^1^ and SPM12^2^ in Matlab (Math Works; http://www.mathworks.com/products/matlab/). To maintain the magnetization balance, we discarded the first 10 functional volumes. Slice timing and realignment for head motion correction were performed. The images were then normalized to the Montreal Neurological Institute template (resampling voxel size = 3 mm × 3 mm × 3 mm) by using the DARTEL method and they were smoothed using a 6-mm full-width at half-maximum (FWHM) Gaussian kernel. Detrending was done to remove linear trends. Lastly, the nuisance covariates, including the 24 head motion parameters and average signals from the cerebrospinal fluid and white matter, were removed by linear regression. The entire dataset of a particular patient was discarded if the maximum value of the head translation (rotation) movement was >2.0 mm (2.0^°^).

#### 2.4.2. ALFF/fALFF calculation

Subsequent to preprocessing, the ALFF/fALFF and FC were calculated. The time courses were converted to the frequency domain by using the fast Fourier transform and the averaged square root of the spectrum across 0.01–0.08 Hz at each voxel was taken as the ALFF. The fALFF analysis was performed following the study by Zou et al. (16). Subsequent to applying a 0.01–0.08-Hz bandpass filter and summing the ALFF values of the fast Fourier transform calculated signal (range, 0.01–0.08 Hz), the sum of the amplitude across 0.01–0.08 Hz was divided by that across the entire frequency range. For standardizing variability across all participants, the mean ALFF was calculated using the formula: ALFF divided by the global mean ALFF; moreover, the mean fALFF was also calculated similarly.

#### 2.4.3. Seed-based FC analysis

Subsequent to the ALFF/fALFF analyses, regions with statistical differences in brain activity were saved as seeds to further investigate the integration of the brain function network via whole-brain FC analysis in a voxel-wise manner. Pearson’s correlation FC analysis was applied to all voxels to obtain an FC correlation coefficient map. Fisher’s z transformation was then performed to allow normal distribution of the data and facilitate statistical analysis.

## 3. Statistical analysis

Demographic and clinical data were analyzed using SPSS 22.0 (Inc., Chicago, IL, United States) statistical software. To compare the differences among the active TAO, inactive TAO, and HC groups, the one-way analysis of variance (ANOVA) test and Kruskal–Wallis test were used for continuous variables with normal distribution; and non-normally distributed data, respectively. Independent-sample t-tests were used for differences between any two groups with continuous variables with normal distribution, and the Mann–Whitney *U*-tests were used for non-normally distributed data. Chi-square tests were used for categorical variables. A *p* value of <0.05 was considered statistically significant.

Between the three groups, The SPM toolbox was used to conduct statistical analyses on ALFF/fALFF and FC. *Post hoc* two-sample t-tests were used to compare values in brain regions between each pair of groups. Significant clusters were identified using a voxel-level threshold of *p* < 0.001, a cluster-level threshold of *p* < 0.05 (Gaussian random field (GRF) correction), and a two-tailed test, and the coordinates were labeled in Montreal Neurological Institute (MNI) space. Pearson’s correlation analysis was performed to evaluate the relationship between the clinical data and ALFF/fALFF and FC values in brain regions with significant group differences. A *p-*value of <0.05 was considered statistically significant. Age, sex, and years of education were adjusted in all statistical models.

## 4. Results

### 4.1. Demographic and clinical characteristics

Table 1 shows the demographic and clinical characteristics of all participants. Between the three groups, there was no significant difference in age (*p* = 0.965), sex (*p* > 0.964), or years of education (*p* = 0.955). The mean CAS was 3.54 ± 1.29 and 1.08 ± 0.96 in the active and inactive TAO groups, respectively (*p* < 0.001). Patients in the active TAO group showed significantly higher TRAb than those in the inactive TAO group (*p* = 0.035). The active TAO group had significantly lower QoL scores for visual function and lid aperture than the inactive TAO group (*p* = 0.032, 0.029). Between the three groups, significant differences were present in the total HDRS (*p* = 0.004) and HARS scores (*p* < 0.001). Between-group comparisons revealed that active and inactive TAO patients had lower visual acuity than HCs (*p* = 0.013). Moreover, the two groups did not have statistically significant differences in terms of visual acuity (*p* = 0.956). There were no statistically significant differences between the three groups in all other clinical characteristics (Table 1).

**Table 1:** Demographic and clinical characteristics of TAO patients and HCs.

Characteristics	AP	IP	HC	*P*-value
Sex (male/female)	12/12	12/14	13/14	0.964
Age (year)	47.42 ± 9.46	47.31 ± 10.71	47 ± 14.77	0.965
Years of education (year)	12.13 ± 4.08	11.92 ± 3.25	12.22 ± 3.46	0.955
Disease duration (year)	18.75 ± 19.95	22.23 ± 28.28	/	0.606
Smoking score	90.83 ± 184.66	64.62 ± 165.09	/	0.766
Antithyroid therapy (yes/no)	17/7	20/6	/	0.624
Restoration of euthyroidism (yes/no)	8/16	13/13	/	0.233
TSH (mIU/L)	1.59 ± 2.09	2.81 ± 4.11	/	0.180
fT3 (pmol/L)	4.33 ± 1.73	3.84 ± 1.4	/	0.207
fT4 (pmol/L)	5.82 ± 7.14	5.74 ± 6.63	/	0.892
TRAb (IU/L)	13.67 ± 14.08	7.23 ± 7.83	/	0.035^*^
Diplopia score	1.71 ± 1.17	1.23 ± 1.25	/	0.163
BCVA	0.84 ± 0.15	0.91 ± 0.14	0.96 ± 0.07	0.013^*^
IOP (mmHg)	20.27 ± 3.77	18.43 ± 3.54	/	0.052
Exophthalmos (mm)	21.69 ± 2.47	19.87 ± 2.8	/	0.058
CAS	3.54 ± 1.29	1.08 ± 0.96	/	<0.001^*^
Lid aperture (mm)	9.94 ± 2.18	10.98 ± 1.65	/	0.029^*^
Eye motility score	1.67 ± 0.47	1.19 ± 0.92	/	0.059
GO-QoL: visual functioning	41.42 ± 26.79	60.36 ± 31.26	/	0.032^*^
GO-QoL: appearance	61.72 ± 25.47	50.86 ± 18.1	/	0.191
HARS	11.54 ± 8.05	10.35 ± 7.12	2.19 ± 2.55	<0.001^*^
HDRS	6.29 ± 5.91	3.58 ± 3.4	2.52 ± 1.03	0.004^*^

Continuous variables are presented as the mean (± standard deviation) or as the median (interquartile range). Categorical variables are presented as the number (%) and counts. TAO, thyroid-associated ophthalmopathy; AP, active TAO patients; IP, inactive TAO patients; HC, healthy controls; TSH, thyroid-stimulating hormone; fT3, free triiodothyronine; fT4, free thyroxine; TRAb, Thyroid-Stimulating Hormone Receptor Antibodies; BCVA, best corrected visual acuity; IOP, intraocular pressure; CAS, clinical activity score; GO-QOL, quality of life in thyroid eye disease; HARS, Hamilton anxiety scale; HDRS, Hamilton depression scale. *P <0.05.

### 4.2. ALFF/fALFF and FC analysis

**Figure 1:**
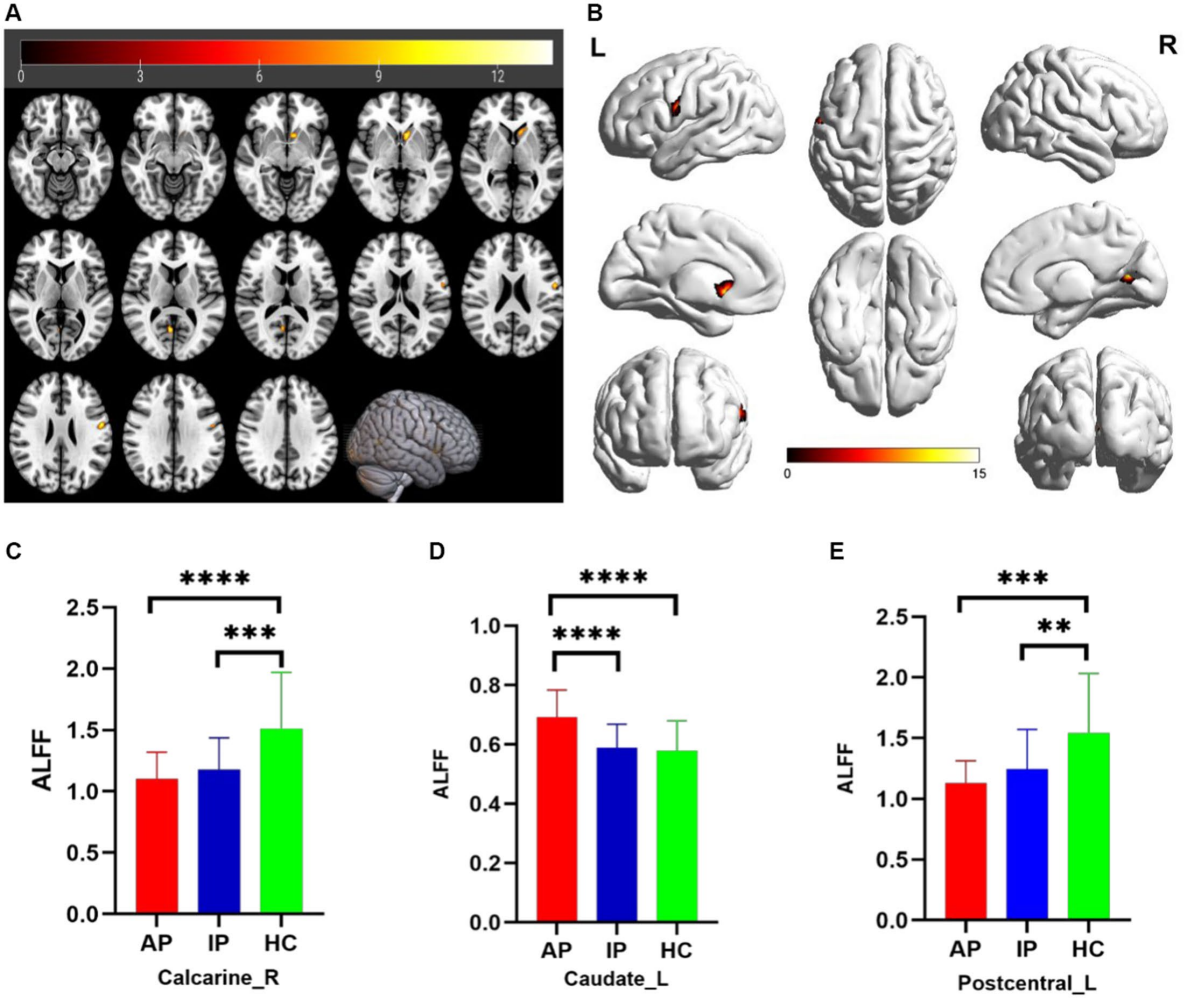
Brain regions with significant differences in ALFF among AP, IP, and HC groups based on one-way analysis of covariance in 2D and 3D picture **(A,B)**. *Post hoc* two-sample *t*-test showing comparisons of ALFF in Calcarine_R, Caudate_L, and Postcentral_L among AP, IP, and HC groups **(C–E)**. AP, active patient; IP, inactive patient; HC, healthy control; ALFF, amplitude of low-frequency fluctuation. **P<0.01, ***P<0.01, ****P<0.001.

**Figure 2:**
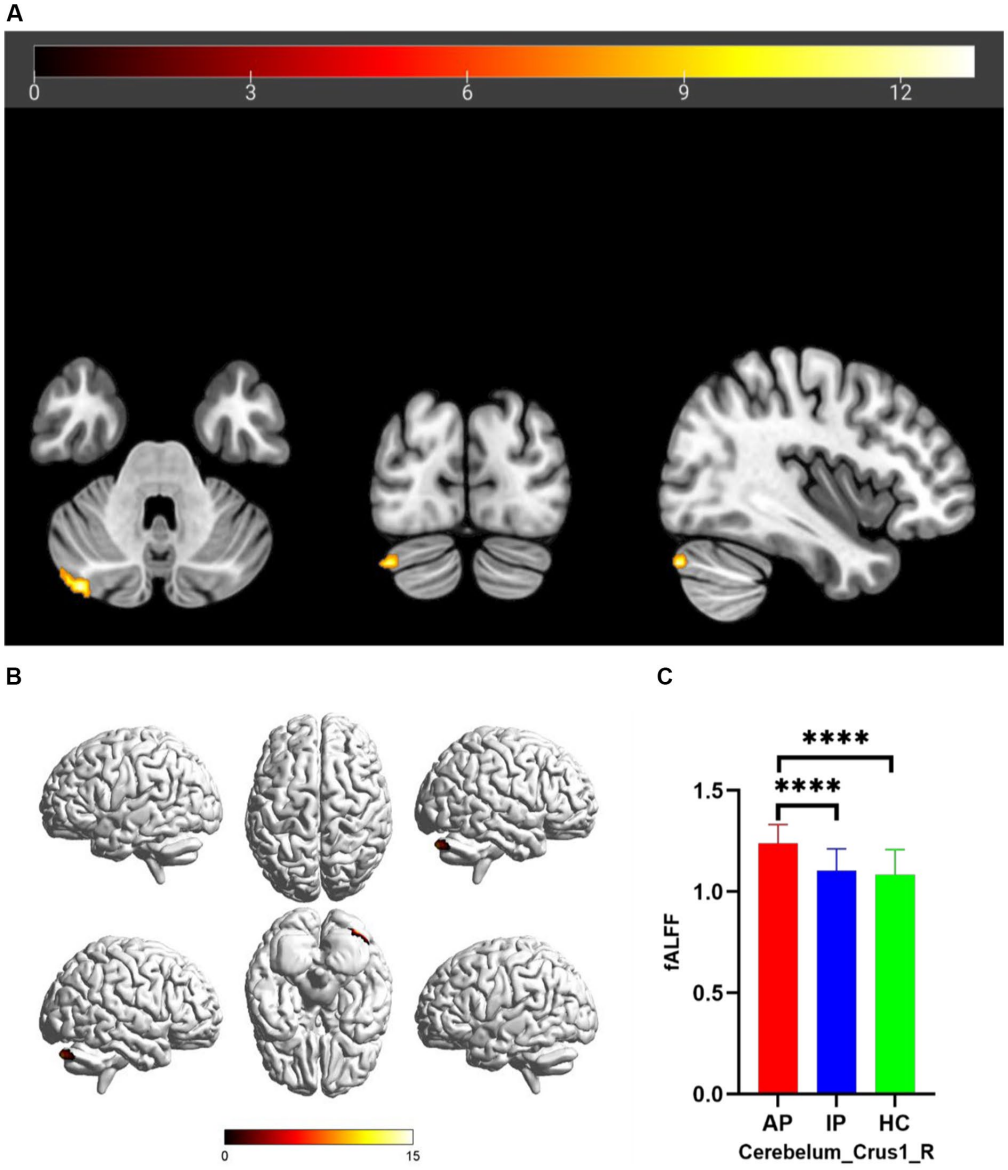
Brain regions with significant differences in fALFF among AP, IP, and HC groups based on one-way analysis of covariance in 2D and 3D picture **(A,B)**. *Post hoc* two-sample *t*-test showing comparisons of fALFF in Cerebelum_Crus1_R among AP, IP, and HC groups **(C)**. AP, active patient; IP, inactive patient; HC, healthy control; fALFF, fractional amplitude of low-frequency fluctuation. ****P<0.001.

**Figure 3:**
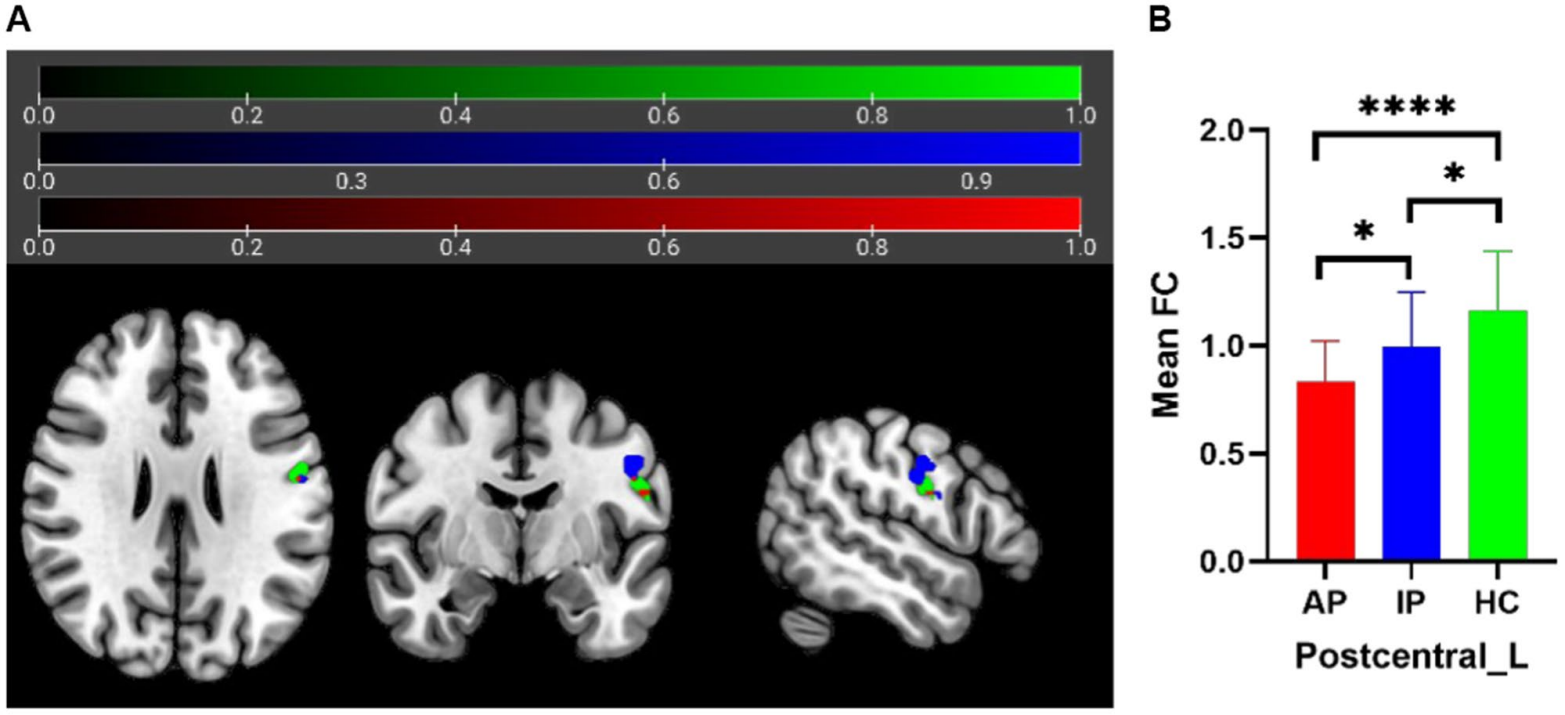
In FC analysis using the Postcentral_L as seed, 2D picture shows significant differences within the Postcentral_L among AP, IP, and HC groups **(A)** (green area: seed; blue area: FC differences in sub-region of Postcentral_L; red area: overlapping region). *Post hoc* two-sample *t*-test showing comparisons of FC within Postcentral_L among AP, IP, and HC groups **(B)**. AP, active patient; IP, inactive patient; HC, healthy control; FC, functional connectivity. *P<0.05, ****P<0.001.

The one-way analysis of covariance (ANCOVA) results showed significant ALFF/fALFF differences among the three groups, primarily in the Caudate_L, Calcarine_R, Postcentral_L, and Cerebelum_Crus1_R regions (voxel *p* < 0.001, cluster *p* < 0.05, cluster level GRF corrected; Table 2). In the pairwise comparisons, both active and inactive TAO groups showed significantly decreased values in the right Calcarine_R and Postcentral_L compare to the HC group (Figure 1). The active TAO group had significantly increased ALFF and fALFF values in the Caudate_L and Cerebelum_Crus1_R, respectively, compared to the HCs and inactive group (Figures 1, 2). We further found that both inactive and active TAO patients exhibited decreased FC in the Postcentral_L compared to HCs, and the active TAO patients exhibited decreased FC compared to the inactive TAO patients (Figure 3).

**Table 2:** Brain areas with significantly different ALFF/fALFF and FC values between groups (voxel *P* < 0.001, cluster *P* < 0.05, cluster-level GRF corrected).

Parameter	Brain regions	F-value	Cluster size	MNI coordinates of peak voxel		
				X	Y	Z
ALFF	Calcarine_R	12.3896	15	6	−63	12
ALFF	Caudate_R	14.0245	37	−9	12	−3
ALFF	Postcentral_L	12.0832	23	−57	−3	21
fALFF	Cerebelum_Crus1_R	14.1279	20	−39	−84	−33
FC	Postcentral_L	12.4138	51	−51	−6	36

ALFF, amplitude of low-frequency fluctuations; fALFF, fractional amplitude of low-frequency fluctuation; GRF, Gaussian random field; MNI, Montreal Neurologic Institute; FC, functional connectivity.

### 4.3. Correlation analysis

**Figure 4:**
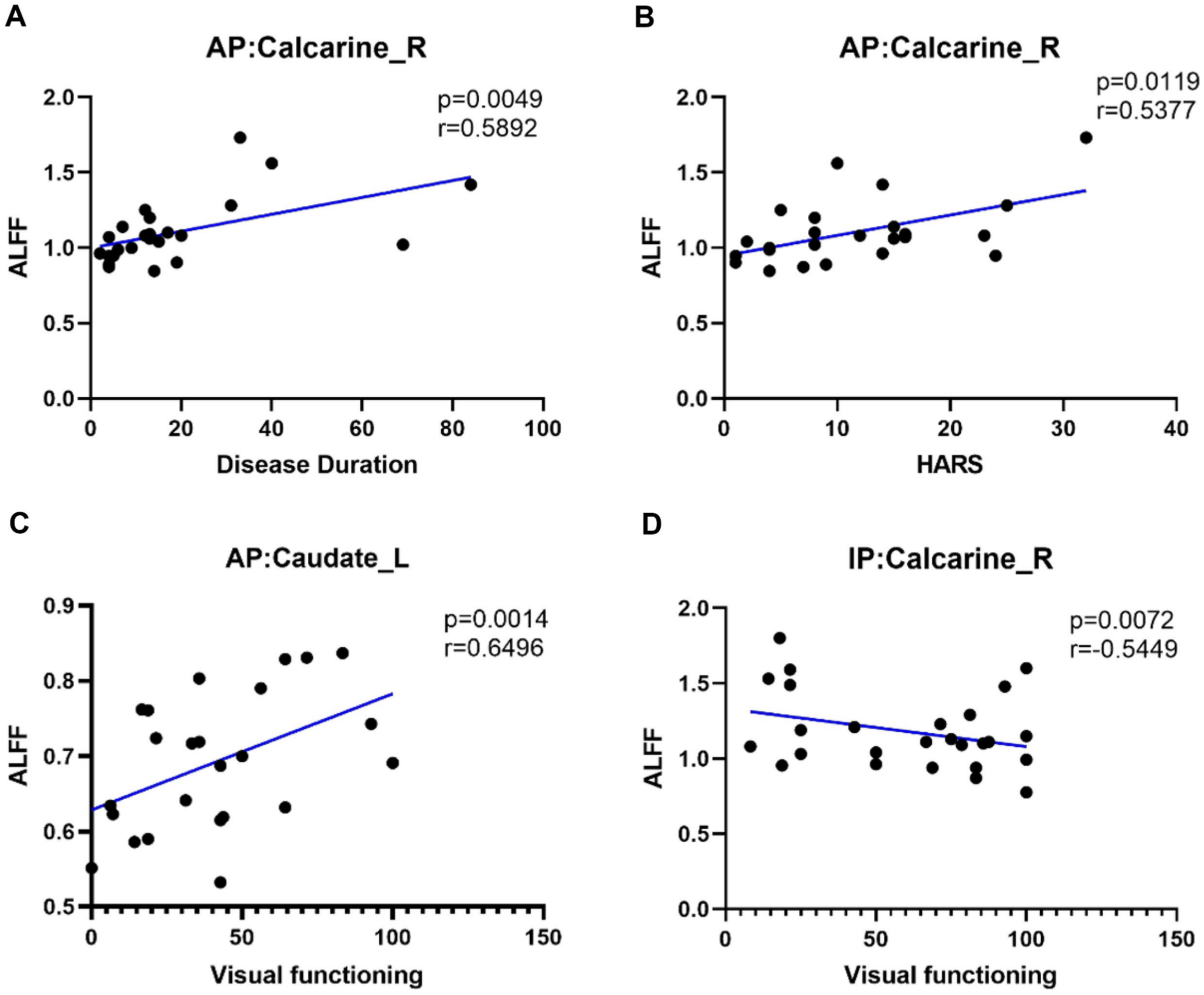
Positive correlation between the ALFF of Calcarine_R and disease duration in AP **(A)**. Positive correlation between the ALFF of Calcarine_R and HARS in AP **(B)**. Positive correlation between the ALFF of Caudate_L and visual functioning in AP **(C)**. Negative correlation between the ALFF of Calcarine_R and visual functioning in IP **(D)**. AP, active patient; IP, inactive patient; HC, healthy control; HARS, Hamilton Anxiety Rating Scale.

In the active TAO group, the ALFF value of the Calcarine_R region was positively correlated with disease duration (r = 0.5892, *p* = 0.0049) and HARS (r = 0.5377, *p* = 0.0119) and that of Caudate_L was positively correlated with visual function (r = 0.6496, *p* = 0.0014). Furthermore, the ALFF value of the Calcarine_R region in the inactive TAO group was negatively correlated with visual function (r = 0.5449, *p* = 0.0072) (Figure 4). No significant correlation was found between the ALFF/ fALFF and FC values with any other clinical parameter.

## 5. Discussion

To our knowledge, this is the first study that systematically investigated the neuropathological mechanisms in active and inactive TAO patients and compared them with HCs by combining ALFF/fALFF and FC measures. This approach allowed the examination of brain alterations from a local-to-global perspective, thereby providing a more comprehensive understanding of TAO.

A previous fALFF study compared TAOs and HCs and found reduced spontaneous neuronal activity in the Calcarine_R in active TAO patients (17). Yet another fALFF study compared TAO patients and HCs and showed reduced activity in the bilateral calcarine brain regions (22). The calcarine is a crucial part of the primary visual cortex and is believed to play a major role in visual processing (23). Moreover, the occipital lobe contains the primary visual area of the brain, and the calcarine is the most reliable anatomic landmark of the medial occipital lobe. Our results showed exclusively reduced ALFF values in the Calcarine_R region of both active and inactive TAO patients that which suggests decreased spontaneous neural activity of the vision-related cortex region, thereby indicating impaired visual information integration. In TAO, chronic organ-specific autoimmune inflammation is present, and the disease course is divided into active and inactive phases. The active phase usually lasts about 18–24 months, and then gradually, the inactive phase begins (24). The results of the correlation analysis showed that increasing Calcarine_R ALFF value in active TAO patients was correlated with prolongation of the disease course; whereas, in inactive TAO patients, increasing ALFF value was noted with decreasing visual function. Thus, these findings indicate impairment in the vision-related brain regions in TAO, and some compensatory mechanisms occur in different individuals with different TAO activity phases. In the active TAO group, individuals with longer disease duration displayed higher ALFF values in the correlation analysis. Furthermore, within the inactive TAO group, individuals with poorer visual functioning exhibited higher ALFF values in the correlation analysis. Of note, although some ALFF compensation occurs in different individuals during the active TAO phase, active TAO has a significantly positive correlation with HARS, and the psychological condition of these patients should be evaluated.

The caudate nucleus is a crucial component of the basal ganglia, traditionally associated with motor processes, emotions, motivation, and cognition. In Parkinson’s disease (PD), the caudate nucleus is of particular interest as it plays a role in cognitive functions such as memory, attention, planning, and skill learning. It also supports strategic planning and the execution of behaviors necessary for achieving complex goals (25, 26). Descending pathways, including the caudate nucleus, also influence eye movements through the superior colliculus (27). In the present study, the ALFF value of the Caudate_L in active TAO patients was significantly higher compared to HCs and inactive TAO patients, which is a finding that has not been previously reported. Based on the functional role of the caudate nucleus, we can speculate that it plays a compensatory role in motor abnormalities caused by eye movement disorders and diplopia, particularly in active TAO patients.

The cerebellum is located in the posterior fossa and plays an important role in visuospatial processing, eye movement, and higher cognitive functions (28). In an fMRI study of patients with exophthalmos of primary hyperthyroidism (EOPH), using a voxel-wise degree centrality (DC) method, the DC value of the cerebellum’s posterior lobe was reduced and negatively correlated with HDRS and HARS (29). Yet another fMRI study of TAO patients and HCs found that TAO patients had higher ALFF values in the left cerebellum, and it was speculated that the elevated intrinsic brain activity in this region reflects functional reorganization to compensate for eye movement abnormalities and visual impairment (15). Although similar changes in ALFF were not found in our study, with a larger sample size and a stricter voxel *p*-value, it was found that the fALFF value of Cecebelum_Crus1_R in active TAOs was significantly increased compared to HCs and inactive TAOs. The most recent part of the cerebellum, particularly crus 1, contributes to parallel cortico-cerebellar loops involved in executive control, salience detection, episodic memory, and self-reflection (30). Our study is consistent with previous reports that suggest abnormal cerebellar activation in TAO patients. Furthermore, we found that this phenomenon was particularly pronounced in active TAO patients.

The postcentral gyrus is a part of the parietal lobe that includes Brodmann areas 1–3 and is located in the primary somatosensory cortex. Brodmann area 3, which is located superior to the postcentral gyrus, processes the sense of touch and pressure from the body’s surface (31). Brodmann areas 2 and 1, which are located inferior to area 3, process more complex sensory information, such as proprioception and fine tactile discrimination (31). These three areas work in conjunction for a detailed and nuanced representation of the body’s sensory experiences. Moreover, they are also closely connected to other brain regions involved in sensory and motor processing, enabling coordinated and efficient movement, and response to sensory stimuli (31). However, there have been no studies investigating whether TAO affects primary sensory cortex function. Only one fMRI study of TAO reported reduced long- and short-range functional connectivity density (FCD) in the bilateral postcentral gyrus (32). We found that Postcentral_L ALFF values in active and inactive TAO patients were lower than those of HCs. Additionally, in the FC analysis using the Postcentral_L as seed, no significant abnormalities in FC between the seed and other brain regions were found; however, abnormalities in the FC within the Postcentral_L were noted. The FC values were decreased, with greater reductions observed in active TAO patients. Numerous connections between sub-regions of the somatosensory cortex have been found (33). Therefore, there is decreased FC between different sub-regions of the somatosensory cortex in TAO patients, indicating the possibility of impaired functional integration. We speculate that this may be due to some TAO symptoms, such as eye pain, pressure sensation, and diplopia, which may affect the patient’s sensory system, leading to decreased ALFF values in the Postcentral_L and decreased internal FC.

This study had several limitations. First, although the sample size was larger compared to previous studies, it is still relatively small. A larger sample size could increase statistical power. Second, more detailed cognitive, memory, and learning-related scales were not used to analyze the correlation of the patient’s symptoms with the abnormal activity regions found in rs-fMRI. Finally, as this was a cross-sectional study, further longitudinal research is needed to further elucidate our understanding of the neural activity changes in TAO patients.

## 6. Conclusion

Our findings demonstrate that the Caudate_L and Cerebellum_Crus1_R, which are primarily related to motor control and coordination and located in the cerebellum and basal ganglia, exhibit significant compensatory mechanisms in active TAO patients. In contrast, the Calcarine_R and Postcentral_L, which are associated with visual and somatosensory cortices located in the occipital and parietal lobes, show varying degrees of impairment in patients with TAO. This research provides valuable insights into the neural etiology of TAO disease, highlighting the presence of brain function abnormalities in affected individuals. These findings underscore the importance of considering the neuropsychological aspects of the disease in treatment planning and management.

## Data availability statement

The raw data supporting the conclusions of this article will be made available by the authors, without undue reservation.

## Ethics statement

The studies involving human participants were reviewed and approved by the Ethics Committee of Shanghai Ninth People’s Hospital, Shanghai Jiao Tong University School of Medicine. The patients/participants provided their written informed consent to participate in this study.

## Author contributions

LZ, HuZ, and XT designed the study. YuL, SW, JQ, and YiL collected the data. YT and YS analyzed the data. MJ and HaZ wrote the manuscript. HuZ, JS, and YiL revised the manuscript. All authors contributed to the article and approved the submitted version.

## Funding

This work was supported by funds from the National Nature Science Foundation of China (91859202 and 82172049).

## Conflict of interest

YS from a commercial company, Siemens Healthineers Ltd., was a MR collaboration scientist doing technical support in this study under Siemens collaboration regulation without any payment and personal concern regarding to this study.

The remaining authors declare that the research was conducted in the absence of any commercial or financial relationships that could be construed as a potential conflict of interest.

The reviewer HL declared a shared affiliation with the authors MJ, HaZ, YuL, SW, JQ, YT, YiL, JS, LZ, HuZ, XT at the time of review.

## Publisher’s note

All claims expressed in this article are solely those of the authors and do not necessarily represent those of their affiliated organizations, or those of the publisher, the editors and the reviewers. Any product that may be evaluated in this article, or claim that may be made by its manufacturer, is not guaranteed or endorsed by the publisher.

## Footnotes

^1^www.restfmri.net

^2^https://www.fil.ion.ucl.ac.uk/spm
